# Confounding Factors Impacting microRNA Expression in Human Saliva: Methodological and Biological Considerations

**DOI:** 10.3390/genes13101874

**Published:** 2022-10-16

**Authors:** Rhea Sullivan, Austin Montgomery, Anna Scipioni, Pooja Jhaveri, Adam T. Schmidt, Steven D. Hicks

**Affiliations:** 1 Department of Pediatrics, Penn State Hershey College of Medicine, Hershey, PA 17033, USA; 2 Department of Obstetrics, Morsani College of Medicine, University of Southern Florida, Tampa, FL 33606, USA; 3 Department of Psychological Sciences, Texas Tech University, Lubbock, TX 79409, USA

**Keywords:** miRNA, salivary, biomarker, housekeeping, stabilizer

## Abstract

There is growing interest in saliva microRNAs (miRNAs) as non-invasive biomarkers for human disease. Such an approach requires understanding how differences in experimental design affect miRNA expression. Variations in technical methodologies, coupled with inter-individual variability may reduce study reproducibility and generalizability. Another barrier facing salivary miRNA biomarker research is a lack of recognized “control miRNAs”. In one of the largest studies of human salivary miRNA to date (922 healthy individuals), we utilized 1225 saliva samples to quantify variability in miRNA expression resulting from aligner selection (Bowtie1 vs. Bowtie2), saliva collection method (expectorated vs. swabbed), RNA stabilizer (presence vs. absence), and individual biological factors (sex, age, body mass index, exercise, caloric intake). Differential expression analyses revealed that absence of RNA stabilizer introduced the greatest variability, followed by differences in methods of collection and aligner. Biological factors generally affected a smaller number of miRNAs. We also reported coefficients of variations for 643 miRNAs consistently present in saliva, highlighting several salivary miRNAs to serve as reference genes. Thus, the results of this analysis can be used by researchers to optimize parameters of salivary miRNA measurement, exclude miRNAs confounded by numerous biologic factors, and identify appropriate miRNA controls.

## 1. Introduction

In the past decade, the number of human saliva biomarker studies have more than tripled. The growing interest is founded upon the fact that biochemical and molecular players involved in disease pathogenesis are robustly detected in a saliva. Inflammatory mediators [1], signaling molecules [2], nucleic acids [3], microbes [4,5], and hormones [6] found in saliva predict disease onset or correlate with disease severity. Further, research consistently demonstrates that salivary biomarkers are indicative of human health beyond the oral cavity [7,8]. Collection of saliva is also a non-invasive and inexpensive way to study systemic processes; parents of child participants in longitudinal cohort studies have been shown to prefer saliva diagnostics compared to other forms of biofluid measurements (e.g., blood draw), enhancing participant enrollment and retention [9]. As the feasibility of saliva diagnostics gains popularity [10], the methodologies for collecting and studying it has varied widely. For example, the growing excitement surrounding saliva miRNA biomarkers has led to a surge in pilot studies, many of which may lack adequate consideration of biologic and methodologic factors with the potential to negatively impact rigor and reproducibility.

miRNAs are small (18–25 bp), non-coding RNAs that repress translation of target messenger RNAs (mRNAs) [11]. The role of miRNAs in epigenetic regulation is functionally conserved from nematodes to humans [12]. miRNAs contain a seed sequence (4–8 bps) that binds to complementary sites in the 3′ untranslated regions of mature mRNAs, leading to mRNA degradation [13]. A single, mature miRNA can regulate hundreds of genes that contain its complementary seed sequence. This unique feature of miRNA physiology highlights how altered miRNA expression can be leveraged to predict how entire signaling pathways may be altered in human disease. miRNAs possess the potential for critical diagnostic, prognostic, and therapeutic value. There are over 2000 known miRNAs, that collectively regulate over 60% of the genome [14]. Interestingly, out of 12 human biofluids profiled, miRNAs have the second highest concentration in whole saliva [15].

Expression studies of miRNAs from human saliva have been increasing over the past decade, across a number of clinical disciplines. Notably, high powered cohort studies identify predictive miRNAs for of autism spectrum disorder [4,16,17], traumatic brain injury [18,19,20,21] endometriosis [22], and a variety of cancers [23,24,25]. Other recent studies include the prediction of alcohol use disorder [26,27], Parkinson’s Disease [28,29], and malignant pleural effusion [30]. Broad commonalities of these studies include saliva collection, saliva storage, miRNA extraction and isolation, and miRNA expression profiling. However, many of these studies differ in the aforementioned methodological steps. For example, some studies report low mapping of miRNAs in whole saliva [31,32], but do not use RNA stabilizer in their collection methods. Previous studies indicate that absence of RNA stabilizer in human saliva collection decreases RNA yield by over 68% [33]. It remains unclear how the presence or absence of RNA stabilizer may differentially impact the abundance of specific miRNAs.

There are several reports of how miRNA expression within a given disease state may depend on participant factors (age [34,35], sex [36,37,38], prandial status [39,40,41], exercise [42,43,44,45], body mass index (BMI) [41,45,46]), and technical factors (method of saliva collection [47,48,49], presence/absence of RNA stabilizer [33,49], aligner technique [50,51]) in the study design. The individual and cumulative effects of these factors on miRNA-based disease prediction models remain unknown. In order to increase generalizability of salivary miRNA-omics studies and reported biomarkers, it is imperative to understand how these seemingly simple factors affect miRNA expression patterns.

Another gap facing the field of salivary miRNA biomarker discovery is the lack of reported endogenous control miRNAs [52]. Historically, the miRNA field normalizes to small nucleolar RNAs, as these have structural roles across cell types. However, depending on the disease being studied or the fluid being collected, small nucleolar RNAs may be as variable as disease-associated miRNA levels [53,54]. An alternative approach is to identify miRNAs with the least variable read counts across all samples; this normalization technique is most common amongst salivary miRNA studies done in disease cohorts [52]. Unfortunately, many cohort studies remain underpowered, and only report “disease-associated” control miRNAs, not healthy participant-associated ones. Further, standard mRNA housekeeping genes in saliva, such as *GAPDH,* may not be optimal controls across certain miRNA cDNA amplification kits [55]. In large sequencing studies, global normalization can be utilized—but this approach does not work for qPCR profiling where a limited number of targets are measured [51]. These challenges, compounded by use of inconsistent sample collection methods, further complicate identification of control miRNAs. These inconsistencies potentiate barriers to developing qPCR-based clinical tests, thereby limiting the wide-spread adoption and investigation of these powerful regulators. Thus, the field of salivary miRNA biomarker research is still in need of robust control miRNAs reported from high powered, healthy participant studies.

Research from our group demonstrates that salivary miRNAs display diurnal oscillations, providing evidence that it is important to control for the time of day in which saliva is collected [56]. However, robust research on other factors that may systematically impact miRNA expression is lacking. In order to further address the need for consistency of other participant and technical factors in salivary miRNA-omics studies, we present a high-powered study that quantifies differences in saliva miRNA expression as a result of 3 technical factors (aligner type, presence/absence of RNA stabilizer, method of saliva collection) and 6 participant factors (sex, age, BMI, prandial status, exercise status, and within-participant variation over time). Further, we present salivary miRNAs that may serve as stable control miRNAs, based on their low computed coefficient of variability across 1225 saliva samples, and their stable expression levels within individuals over time.

## 2. Materials and Methods

### 2.1. Participants

This retrospective cross-sectional study utilized 1225 saliva samples from 922 individuals who were enrolled as control participants in parent studies of five medical conditions: adverse childhood events (*n* = 48), anorexia nervosa (*n* = 117), eosinophilic esophagitis (*n* = 100), infant allergies (*n* = 233), or mild traumatic brain injury (*n* = 727). For the current analysis, no samples from individuals with acute medical conditions were utilized.

### 2.2. Data and Sample Collection

Participants self-reported age, sex, and race. Time and date of sample collection were also recorded, and BMI was abstracted from the electronic medical record. Saliva samples were obtained between January 2017 and May 2021. Two variables related to sample collection were recorded for each saliva sample: collection technique (swab versus expectorant), and presence or absence of RNA stabilizing solution. Saliva swabs (*n* = 991) were obtained by applying ORE-100 devices (DNA Genotek, Ottawa, ON, Canada) to the sublingual and parotid regions for approximately 20 s, and expectorated samples (*n* = 234) were obtained through active expectoration of 1 mL using RE-100 devices (DNA Genotek, Ottawa, Canada). 1125 samples were collected with Oragene RNA stabilizer while 100 were collected without stabilizer. Three hundred and three participants provided repeat samples: there were 58 participants who provided samples in a fasting and non-fasting state [41], 93 participants who provided samples pre/post-exercise, and 152 participants who provided baseline samples two to six months apart [57].

### 2.3. Sample Handling and RNA Processing

All samples were handled per manufacturer instructions. Samples were stored at −80 °C within eight weeks of collection and underwent only one freeze–thaw cycle prior to RNA extraction with the miRNeasy kit (Qiagen, Inc., Germantown, MD, USA). RNA quality was confirmed with an Agilent Bioanalyzer 2100 (Agilent, Santa Clara, CA, USA) prior to RNA sequencing. Sequencing occurred at the SUNY Upstate Molecular Core Facility (Syracuse, NY, USA) with the Illumina TruSeq Small RNA Prep protocol and a NextSeq500 instrument (Illumina; San Diego, CA, USA). A targeted read depth of 10 million paired-end reads was utilized for all samples. FASTQ files for each sample were aligned to miRBase22 using either the Bowtie 1 or Botwie2 aligner in Partek Flow. Read quality score and total read count were used to ensure RNA sequencing quality. Principal component analysis was used to examine the dataset for sphericity and identify sample outliers. For comparing miRNA expression between aligners, 529 overlapped and thus were subsequently analyzed within-subjects. For all other analyses, miRNA reads aligned with Bowtie1 were used. The 643 miRNA features detected in Bowtie1 aligned reads (raw read counts ≥ 10) in more than one sample were quantile normalized and auto-scaled (mean-centered and divided by the standard deviation) prior to statistical analysis. For each miRNA feature, prevalence (number of samples detected), median read count, sum of total counts, percentage of total counts, and coefficient of variation was calculated.

### 2.4. Statistical Analysis

There were 643 miRNAs analyzed in all comparisons, except between alignment methods. Between Bowtie1 and Bowtie2, 529 miRNAs overlapped and were subsequently analyzed. A Wilcoxon Rank Sum test was used to compare the 643 miRNAs across two pre-analytic variables (collection technique, and presence/absence of RNA stabilizer), and one post-analytic variable (alignment tool). The effect of each technical variable on total miRNA profile was visualized with a two-dimensional partial least squares discriminant analysis (PLS-DA) in MetaboAnalyst 5.0 [58]. Next, the effect of participant factors on saliva miRNA profiles was assessed using either Spearman Rank Correlation testing (age, BMI), or Wilcoxon Rank Sum testing (sex). Finally, within-subject non-parametric tests were used to identify the impact of food intake, exercise, and within-participant variation over time on saliva miRNA profiles. For all tests, the miRNAs with log2 (Fold Change) > 2.0 and a false detection rate (FDR) corrected *p*-value < 0.05 were considered to display a significant effect.

## 3. Results

### 3.1. Patient Demographics and Sample Attributes

Patient demographics are listed in Table 1. A total of 922 healthy participants were studied. Most of the participants were pediatric, though ages ranged from 1 month-old to 39 years (Table S1). A total of 1225 saliva samples were collected, with 303 participants contributing repeat samples. All sample characteristics, including aligner type, RNA stabilizer, collection method, prandial status, and exercise status are shown in Table 2.

### 3.2. Salivary miRNA Expression Results

In Bowtie1-aligned samples, 2653 different miRNAs were detected with at least one read count per sample. There were 643 miRNAs detected with at least 10 read counts in at least one sample. There were 324 miRNAs with reliable presence (≥10 read counts in at least 10% of samples). The most commonly detected miRNA was miR-27b-3p, present in 1169 out of the 1225 samples. miR-27b-3p also had the most abundant read count out of all miRNAs, with a total raw read count number of 40,926,803 across the 1225 samples (average of 26,415 counts per sample). Interestingly, 25 miRNAs account for over 80% of all miRNA read counts across all saliva samples (Table 3). All miRNA expression data is located in Table S1.

### 3.3. miRNA Expression Variation as a Result of Technical Factors: Overview

**Figure 1:**
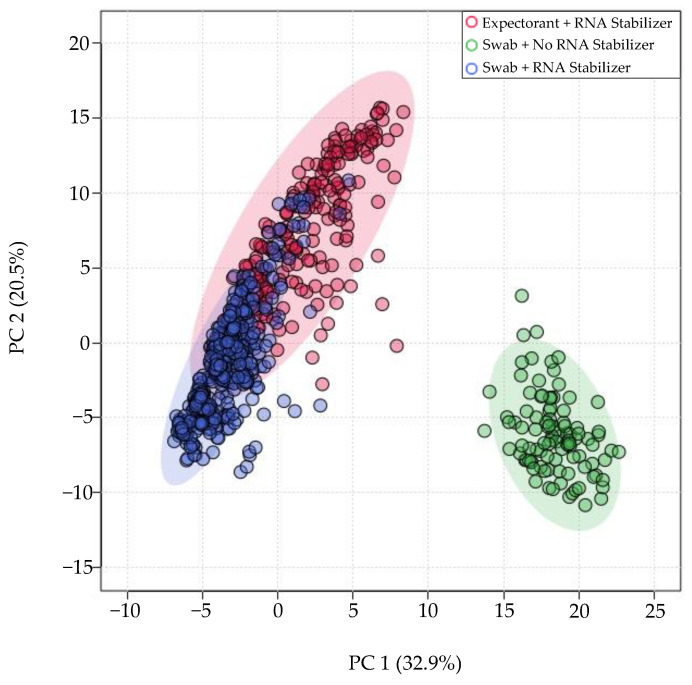
Differences in RNA stabilizer introduce greater miRNA expression variation than differences in method of collection. PCA plot of samples collected with expectorant and RNA stabilizer (red), swab and no RNA stabilizer (green), and swab with RNA stabilizer (purple).

miRNA profiles of all samples aligned with Bowtie1 underwent multivariate dimension reduction using 2D PCA across 2 classes of technical factors: method of saliva collection (swab vs. expectorant), and the presence/absence of RNA stabilizer (Figure 1). The effect of these technical factors explained 32.9% (PC 1) of the miRNA expression variance. Most noticeably, differences in RNA stabilizer (presence vs. absence) had the greatest effect on expression variance. A moderate effect of method of collection (swab vs. expectorant) was also seen in the Bowtie1 aligned samples.

### 3.4. Individual Effects of Aligner Tool, Saliva Collection Method, and Presence/Absence of RNA Stabilizer on miRNA Expression

**Figure 2:**
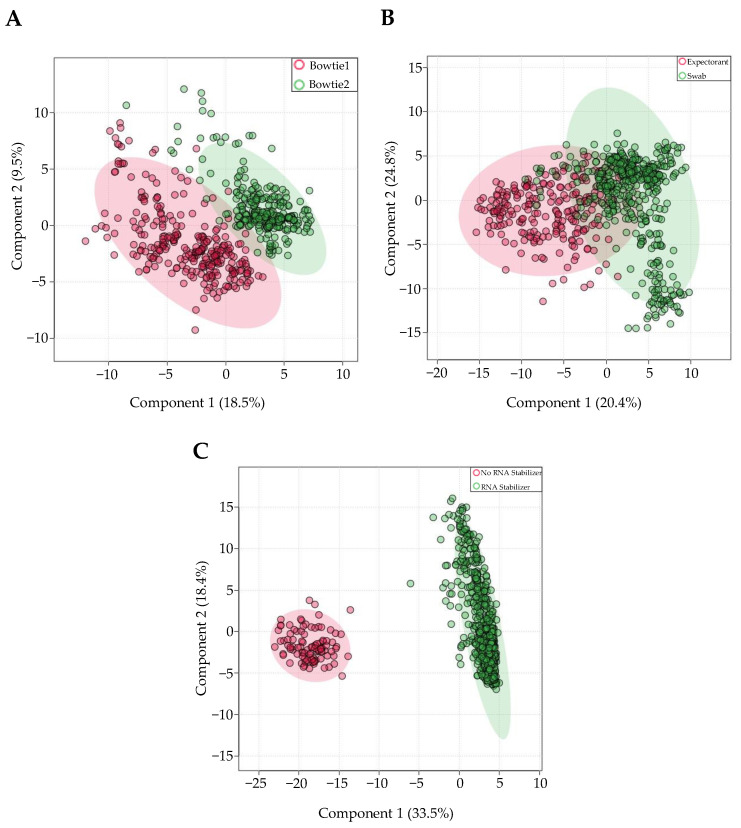
Salivary miRNA expression is affected by differences in three technical factors. PLS-DA plot of 291 samples that were aligned with both Bowtie1 and Bowtie2 for within subject comparison (**A**). For PLS-DA plots visualizing differences in methods of collection (**B**) and use of RNA stabilizer (**C**), 734 samples were used.

Samples from unique individuals (*n* = 291) were aligned with both Bowtie1 and Bowtie2 aligners within Partek-Flow software. Of all the mapped miRNAs, 529 overlapped between Bowtie1 and Bowtie2, and were subsequently analyzed. Within-subjects comparison using Wilcoxon-rank sum tests showed that only 23 miRNAs out of 529 miRNAs were differentially expressed (*FDR* < 0.05 and |log_2_(FC)|>2) in the Bowtie1-aligned samples, compared to the Bowtie2-aligned samples. All 23 miRNAs were increased in Bowtie1 aligned samples compared to Bowtie2-aligned samples. miR-203b-5p had the greatest significant increase in Bowtie1-aligned samples (*FDR* = 3.47 × 10^−94^, log_2_(FC) = 15.30). PLS-DA revealed that differences in aligner type explain 18.5% (Component 1) of the variance in miRNA expression across all samples (Figure 2A).

Next, 734 samples from unique individuals were stratified by method of saliva collection. Out of the 643 miRNAs consistently present in saliva, 161 were differentially expressed in swab-collected samples, relative to expectorated samples. PLS-DA revealed that differences in sample collection contributed 24.8% of variance in miRNA expression (Component 2) (Figure 2B). There were 161 miRNAs that were differentially expressed between groups, with 123 miRNAs increased in swab samples relative to expectorated samples. Interestingly, there is a population of miRNAs that increased in swab samples by over 8-fold, including miR-21-5p, miR-191-5p, miR-148b-3p, miR-12136, and miR-146b-5p. Notably, swab samples had higher average read counts (350,261 +/− 310,066) than expectorant samples (48,706 +/− 196,060; *p* < 0.0001), reflecting an 86% increase in read counts of swab-collected samples.

Next, the same 734 samples were stratified by the presence or absence of RNA stabilizer within the saliva collection vessel. 300 out of 643 miRNAs were differentially expressed in the presence of RNA stabilizer, relative to the absence of RNA stabilizer. There were 118 miRNAs with a relative increase, and 182 miRNAs with a relative decrease. PLSDA revealed that differences in RNA stabilizer accounted for 33.5% (Component 1) of the variability in salivary miRNA expression (Figure 2C). miR-2054 and miR-1915-5p had the two most significant increases with RNA stabilizer, with a fold change of 15.18 (*FDR* = 9.99 × 10^−57^) and 13.85 (*FDR* = 9.99 × 10^−57^), respectively. Most notably, there was a 98% decrease in total read counts in samples without RNA stabilizer. There was an average of 3.62 × 10^5^ +/− 3.59 × 10^5^ read counts in samples with RNA stabilizer and an average of 6.67 × 10^3^ +/− 5.12 × 10^3^ reads in samples without RNA stabilizer.

### 3.5. There Is Minimal Sex-Specific miRNA Expression Variation in Saliva

**Figure 3:**
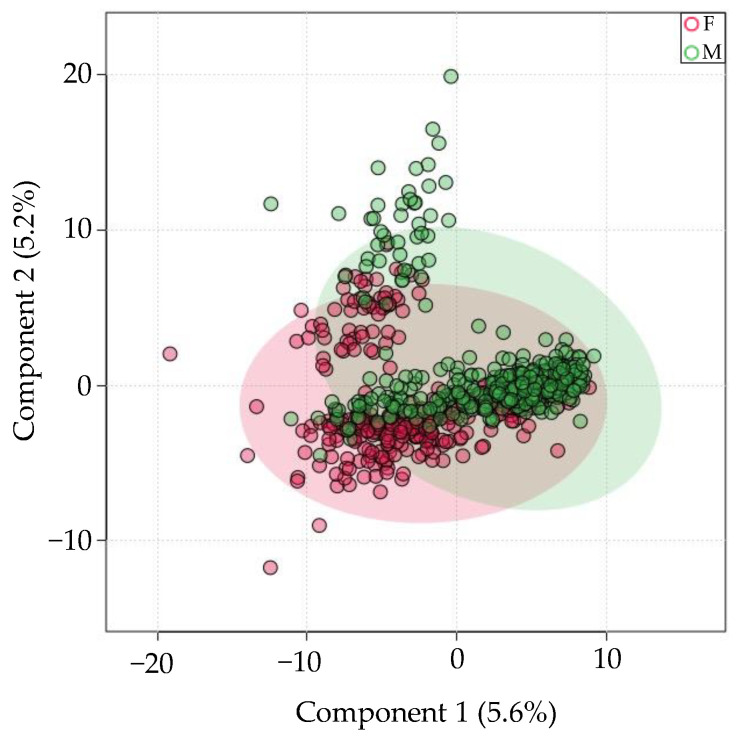
There is minimal sex-specific miRNA expression variation in saliva. PLS-DA-plot of the most consistently present miRNA in saliva, grouped by female (F, red) or Male (M, green) sex.

Next, 855 samples from unique individuals were stratified by sex. Out of the 643 miRNAs consistently present in saliva, only 10 were differentially expressed. PLS-DA revealed high class overlap between samples (Figure 3). PC1 accounted for only 5.6% of the variation. miR-1468-5p had the great fold-change in females relative to males (log_2_(FC) = 6.58, *FDR* = 0.0007). All significantly different miRNAs were upregulated in females relative to males.

### 3.6. Differences in Prandial Status at the Time of Saliva Collection Account for Moderate Variation in miRNA Levels

**Figure 4:**
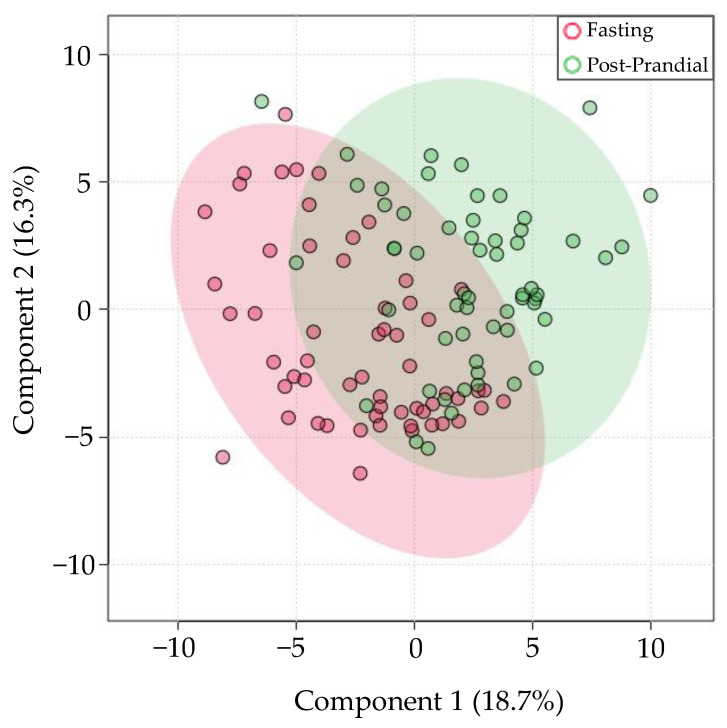
Differences in prandial status are responsible for a moderate amount of saliva miRNA variation. PLS-DA scores plot between groups fasting (red) and post-prandial (green) samples.

Next, 116 samples from 58 healthy participants were stratified into “fasting” and “post-prandial” samples (obtained after over-night fast, and 15 min after breakfast, respectively). All samples were collected by expectoration and aligned with Bowtie1. Caloric intake resulted in differential expression of 11 out of the 643 miRNAs. The PLS-DA scores plot shown in Figure 4 shows that differences in prandial status may explain 18.7% of miRNA expression variation. miR-6748-3p had the most significant increase in post-prandial samples, relative to fasting samples (*FDR* = 8.43 × 10^−6^, log_2_(FC) = 9.19). Other salivary miRNAs significantly affected by prandial status include miR-4451 and miR-657.

### 3.7. Exercise has Minimal Impact on Saliva miRNA Profiles

**Figure 5:**
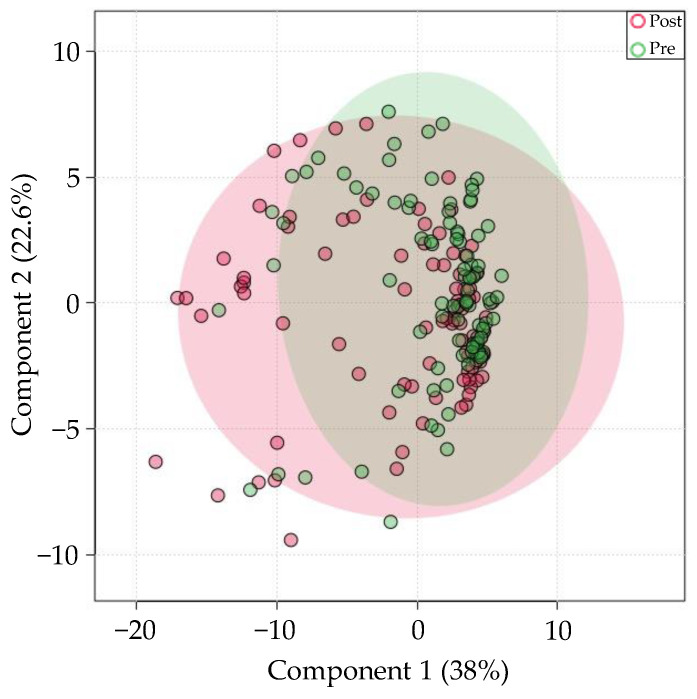
Differences in exercise state confer minimal miRNA variation. PLS-DA plot of pre- (green) and post-exercise (red) samples.

To identify how salivary miRNA levels changes with exercise, 93 collegiate athletes provided 186 saliva samples 15 min before and after participation in a variety of sports (e.g., soccer, lacrosse, rowing, distance running, football). Of all 643 miRNAs consistently present in saliva, only 1 was differentially expressed between groups. PLS-DA revealed high overlap between groups (Figure 5). miR-3689f was the lone miRNA that was significantly increased post-exercise, relative to the pre-exercise group (*FDR* = 0.029, log2(FC) = 2.1).

### 3.8. miRNA Expression Indicates over 75% of miRNAs Consistently Present in Saliva Are Associated with BMI and Age

**Figure 6:**
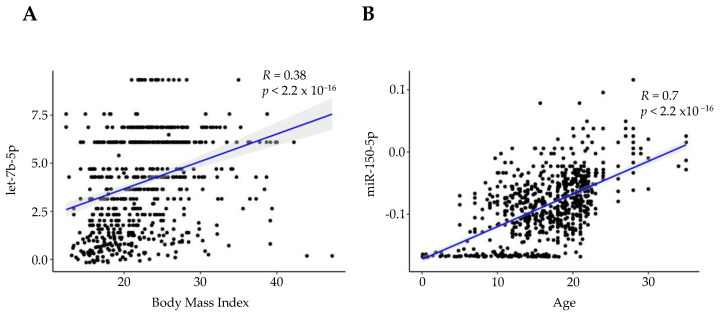
Top salivary miRNAs that correlate with participant BMI and age. Spearman rank correlations between top saliva miRNAs and BMI (**A**) and age (**B**).

The association between miRNA levels and BMI was interrogated using saliva samples from 873 unique participants. Out of the 643 miRNAs consistently present in saliva, 524 were associated with BMI, however none had an |*R*| greater than or equal to 0.5. The miRNA with the strongest direct correlation with BMI was let-7b-5p (*R* = 0.38, *FDR* = 6.901 × 10^−22^) (Figure 6A). Next, associations between miRNA levels and age were explored using saliva samples from 914 participants (range 0.08–35 years of age, mean age = 14.27 years). Out of the 643 miRNAs consistently present in saliva, 517 were associated with age. There were 114 miRNAs that displayed an |*R*| greater than or equal to 0.5. The miRNA most correlated with age was miR-150-5p (*R* = 0.7, *FDR* = 6.37 × 10^−125^) (Figure 6B). Correlation coefficients of miRNAs are presented in Table S1.

### 3.9. Intra-Individual Salivary miRNA Levels Are Relatively Stable over Time for Adults, But Not Infants

**Figure 7:**
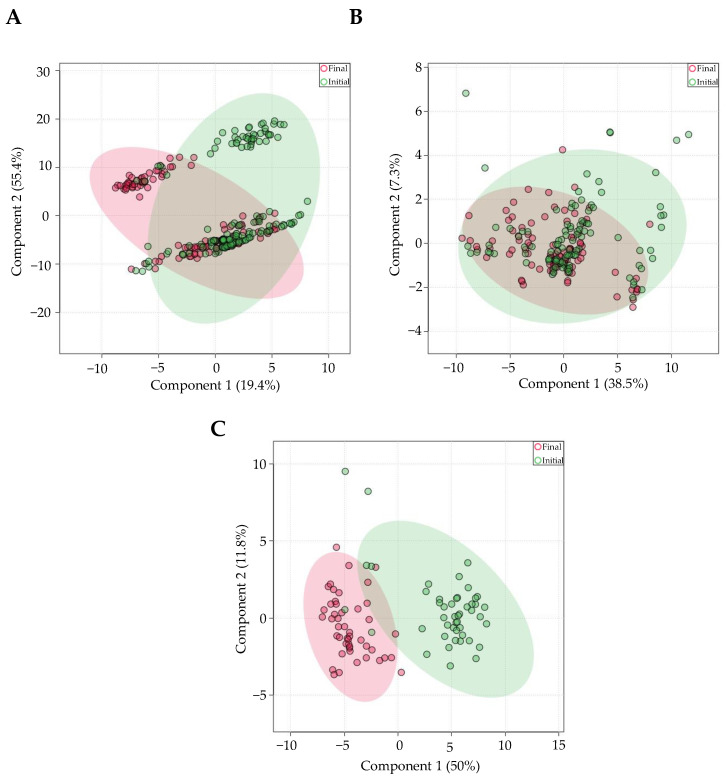
Intra-participant miRNA expression variability is greater in infants compared to young adults. PLS-DA revealed 3 distinct groups between time points when all samples are combined (**A**). When stratified by age, high miRNA expression overlap was shown in young adult samples (**B**), whereas distinct variation is shown between infant time points (**C**).

Repeat saliva samples taken 1–6 months apart from 152 individuals were compared for changes in miRNA levels. PLS-DA revealed three distinct groups (Figure 7A). The samples were chosen to be further stratified by age, since 94 of the samples were from infants (mean 0.123 +/− 0.06 years), and 210 samples were from young adults (mean 22.45 +/− 4.45 years). There was minimal miRNA expression variation in young adults, as seen with the high degree of overlap on PLS-DA analysis (Figure 7B). Only 7 miRNAs (out of 643) differed between the initial and final sample. In young adults, miR-6765-3p had the most significant change within participants from the initial to final saliva sample (*FDR* = 0.0379, log2(FC) = 3.852). Interestingly, PLS-DA revealed distinct miRNA expression differences in the infant age group (Figure 7C). Out of the 643 miRNAs most commonly present in saliva, 50 were significantly different between the initial and final saliva samples. miR-320d had the most significant change between samples (*FDR* = 2.28 × 10^−12^, log_2_(FC) = 3.26).

### 3.10. miRNAs as Internal Control Genes

To address the lack of reported salivary miRNA “control genes” in the scientific literature, we calculated the coefficient of variation for all 643 consistently present miRNAs, across 1225 samples. miR-345-5p had the lowest coefficient of variation of 76.76. Other miRNAs with relatively low coefficients of variation included let-7d-3p (77.6), miR-421 (82.6), and miR-589-5p (83.5). Only 4 miRNAs displayed a coefficient of variation below 90 and were present in over 95% of samples: miR-25-3p (80.9) miR-16-5p (84.3), miR-23a-3p (84.7), and miR-186-5p (89.1). All miRNA expression data, along with respective coefficients of variation, are reported the Table S1.

## 4. Discussion

**Figure 8:**
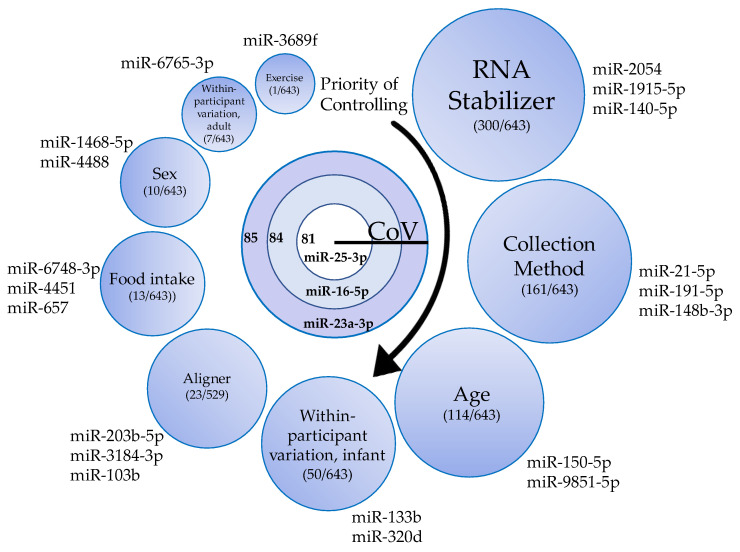
Top “control miRNA” candidates and ranked confounders for consideration. The top three control miRNA candidates identified by coefficient of variation (CoV) and abundance (center). Technical and biologic variables that may impact miRNA analyses are rank-ordered based upon the number of miRNAs differentially expressed between conditions (except age and BMI, where rank is based on the number of miRNAs with an |R| ≥ 0.5). Zero miRNA correlated with BMI with an |R| greater than or equal to 0.5. All miRNAs listed outside of the variable circles are the top significantly different miRNA between conditions.

The current study used a high-powered approach to quantify the effect of controllable factors on salivary miRNA expression variation. We investigated three technical factors: aligner type (Bowtie2 vs. Bowtie1), RNA stabilizer (presence vs. absence), and method of collection (swab vs. expectorant). We also examined biological factors affecting cohort design, including age, caloric intake, sex, exercise, and within-participant variation over time. The most important variables, in order of importance (ranked by number of miRNAs significantly altered between groups or number of miRNA with an |*R*| greater than or equal to 0.5) are RNA stabilizer, collection method, age, within-participant variation (infants), aligner, caloric intake, sex, within-participant variation (adults), and exercise. Participant BMI did not correlate with any miRNAs with an |*R*| greater than or equal to 0.5 (Figure 8).

Differences in technical factors, such as RNA stabilizer (300/643 miRNA altered) and collection method (161/643 miRNAs altered), had seemingly larger impacts on miRNA expression than type of aligner used (23/529 miRNAs altered). The former two analyses were between-participant comparisons, whereas the latter comparison was a within-subjects comparison. Within-subject comparison is a powerful approach to mitigate biological noise between participants. Thus, while aligner differences may impact fewer miRNAs than other technical factors, it should be recognized that between-subjects analyses may cloud true variable-specific differences. Regardless, technical factors can be easily controlled by holding these variables constant throughout a study. Given the impacts of differences in RNA stabilizer (presence vs. absence), method of collection (swab vs. expectorant), and aligner (Bowtie1 vs. Bowtie2) on miRNA variation, they should be held constant in every experimental design.

Out of all the biological variables relating to cohort design, age had the largest effect on miRNA expression. Over 500 miRNAs correlated with both age and BMI, and 114 of these had a strong correlation (|*R*| > 0.5, *FDR* < 0.05) with age. Zero miRNAs correlated with BMI above this threshold. Biologic variables appear to have less of an impact on miRNA levels relative to technical factors. However, it should be noted that biological noise was minimized using a within-subjects statistical approach for most analyses for biological factors. Factors of age, caloric intake, sex, within-participant variation, exercise and BMI should still be considered in all experimental studies. This can be done by matching controls, including biologic factors as covariates in regression models, and by avoiding miRNA biomarkers that highly vary with biologic factors (Figure 8). We have provided a list of how many factors affect each salivary miRNA and quantified the significance of the effect in Table S1.

### 4.1. Prevalence Analysis

miR-27b-3p was the most prevalent miRNA in saliva, and accounted for 10% of all miRNA read counts. miR-27b-3p is expressed by many tissues and cell types, though it has been mostly profiled in cancers (i.e., colorectal [59,60], esophageal [61,62], gastric [63,64] and endometrial cancers [65,66]). Broad functions of miR-27b-3p include roles in cell proliferation [67,68] and regulation cell adhesion (i.e., E-cadherins, N-cadherins, ZO-1) [61,69]. Thus, it makes sense that this miRNA would be highly expressed in an area of the body with high epithelial cell turnover [70].

### 4.2. Aligner

It is well known that differences in mRNA expression can be attributed to differences in aligner, yet to our knowledge, the effect of those differences have not been previously quantified in salivary miRNA studies. In this study, differences between Bowtie1 and Bowtie2 aligners could explain up to 18.5% of miRNA variation. Bowtie1 and Bowtie2 are popular alignment tools, given their accuracy for mapping small RNAs [50]. While both utilize the full-minute index [71], based on the Burrows-Wheeler Transform [72], they have important differences. Bowtie1 is an un-gapped aligner, meaning it will fail to align reads spanning gaps. Bowtie2 closes these gaps through single-instruction multiple-data (SIMD) parallel processing. This step allows Bowtie2 to handle a larger range of read lengths [73], whereas Bowtie1 was developed to align shorter reads (25 bp to 50 bp). Additionally, Bowtie2 supports local read alignment, a more accurate method than Bowtie1′s default end to end alignment. Differences in miRNA alignment have been previously reported between these two aligners. Specifically, Bowtie2 “very sensitive local” settings have been reported as the most accurate mapper for miRNAs, though Bowtie1 “best strata” method has also been ranked highly for aligning multi-mapped reads [50]. It should be verified that similar differences in salivary miRNA expression persist with use of other aligner tools, such as STAR.

### 4.3. RNA Stabilizer

It has not been reported which specific miRNAs are most affected by the absence of RNA stabilizer. We found that the miRNAs that required the presence of RNA stabilizer for reliable detection (had most significant fold changes) included miR-2054, miR-1915-5p, and miR-140-5p. Recently, miR-140-5p was identified as a salivary miRNA biomarker for periodontitis. Interestingly, authors reported that miR-140-5p was selectively enriched in saliva exosomes, but not whole saliva collected without RNA stabilizer. One reason for this observation could be that like RNA stabilizer, exosomes protect miRNA cargo in saliva from RNAses and pH-changes [74]. Previous studies have shown that absence of RNA stabilizer can decrease RNA yield by over 68% [33]. We found that whole saliva miRNA read counts measured across all samples decreased by 98% in samples without RNA stabilizer. Further, over 18% of miRNAs consistently present in saliva increased (log2(FC) > 2.0, *FDR* < 0.05) in expression with the presence of stabilizer. These results highlight the importance of using RNA stabilizer in saliva collection kits to increase the yield of miRNA. Most importantly, consistent methods should be used in miRNA biomarker studies, as 46% (300/643) of miRNAs were affected by differences in RNA stabilizer.

### 4.4. Method of Collection

We also report the first quantifiable differences in salivary miRNA expression between methods of collection. As shown in Table S1, there is a population of highly upregulated miRNAs (log_2_(FC) > 7) in samples collected with swab. miR-21-5p had one of the most significant and greatest fold change increases out of all miRNAs profiled in swabbed samples relative to expectorant samples. miR-21 was one of the first oncomiRs discovered and was found to be upregulated in a variety of cancers. Its relevant targets include *PTEN*, *PDCD4*, and *STAT3* [75]. Recently, miR-21 has been identified as a saliva prognostic biomarker of cervical lymph node metastasis in patients with oral squamous cell carcinoma [76]. Consistent methods of saliva collection should be followed when profiling miRNAs of interest, especially when studying miRNAs with significant, large fold changes between differences in collection method, as listed in Table S1.

### 4.5. Sex-Specific Differences on Salivary miRNA Expression

Differences in participant sex had only a small effect on salivary miRNA expression, as measured by PLS-DA (5.6% PC1). Despite minimal variation introduced by sex, miR-1468-5p was most affected with highest expression in female participants. miR-1468-5p is located on the X-chromosome; X-linked miRNAs have previously been associated with sex-specific differences in disease states, specifically rheumatoid arthritis [77]. miR-1468-5p has been shown to directly target the rate-limiting enzyme of nucleotide synthesis, ribonucleotide reductase [78]. Inhibitors of ribonucleotide reductase have been used to treat myeloproliferative disorders, psoriasis, and cancer—though some data show potential use for other autoimmune disorders [79]. It’s known that the highest genomic distribution of miRNAs is on the X-chromosome [80], though more research is needed to understand the role of X-linked miRNAs in diseases with sex biases.

### 4.6. Prandial Status Differences on miRNA Expression

Many studies mention prandial status of participants (fasting vs. post-prandial) for concern of sample contamination with food. However, it remains unknown how specific miRNAs may be altered by the biologic response to a fasting or fed state. miR-6748-3p was most affected by food intake, with high levels in post-prandial samples. Interestingly, miR-6748-3p is predicted to target fatty acid binding protein 5 (*FABP5*) [81], which has roles in fatty acid uptake, transport and metabolism. Additionally, polymorphisms in *FABP5* are associated with type 2 diabetes mellitus [82]. We observed an indirect correlation of miR-6748-3p with BMI (R = −0.25, *FDR* = 7.08 × 10^−10^), but very few patients of this healthy patient cohort were overweight or obese. Another miRNA that was significantly altered by food intake was miR-657 (*FDR* = 0.00188, log2(FC) = 7.3), which has been reported to bind to the 3′ untranslated region of the insulin growth factor 2 receptor (*IGF2R*) [83]. miR-657 is also involved in regulating the inflammatory response in gestational diabetes mellitus [84,85]. Because food intake alters these miRNAs involved in metabolism and inflammation, it is of utmost importance to continue to control for fasting/fed-state at the time of saliva collection.

### 4.7. Exercise Differences on miRNA Expression

miR-3689f was the only miRNA significantly altered by exercise, with low expression post-exercise. Its predicted targets include myotubularin related protein 12 (*MTMR12*) and myosin heavy chain 11 (*MYH11*). *MTMR12* is required for skeletal muscle maintenance [86], while *MYH11* functions as a contractile protein in smooth muscle [87]. This is the first report to show that exercise minimally affects salivary miRNA expression variation. Considering there are many disciplines investigating sports-related injury using saliva miRNA biomarkers, it is beneficial to know that exercise state will likely have minimal effects on miRNA variability.

### 4.8. miRNAs That Correlate with Age and BMI

miR-150-5p had the greatest Spearman rank correlation with participant age. miR-150-5p has been shown to regulate tumor initiation in a variety of cancers [88]. It directly represses TP53 in colon adenocarcinoma to regulate proliferation, cell arrest, and apoptosis [89]. Additionally, miR-150-5p promotes pathologic angiogenesis in age related macular degeneration in a *VEGF*-independent manner [90]. Let-7b-5p had the strongest Spearman correlation with BMI. Let-7b-5p has been previously identified as a plasma biomarker of insulin resistance [91]. As mentioned previously, few samples were derived from patients with high BMIs. This is a limiting factor that could have affected the total number of miRNAs that correlated with participant BMI, or the strength of the Spearman rank correlation. While over 75% of miRNAs consistently present in saliva correlated with both age and BMI, a higher number of miRNAs had stronger correlations with age (|R| > 0.5). Both biological variables should be controlled through participant matching and/or inclusion as covariates in regression models.

### 4.9. Variation of Intra-Participant Salivary miRNA Expression Is Greater in Infants Than Young Adults

We reported that a majority of miRNAs in saliva are impacted by age, but that analysis did not account for intra-participant variability over time. Intra-participant miRNA expression had greater variation in infants than in young adults in repeat samples taken 1–6 months apart (Figure 7). miR-133b had the greatest fold change (log2(FC) = 9.8, *FDR* = 2.17 × 10^−10^) and miR-320 had the most statistically significant change (log2(FC) = 3.2, *FDR* = 2.2 × 10^−12^) in final samples compared to initial samples in infants. Interestingly, miR-133b plays an important role in the development of human skeletal muscle and myofiber organization [92]. Additionally, miR-133b was shown to increase with age in mouse muscle-derived exosomes from 2 weeks to 1 year of life [93]. Because these samples were taken from 1-month and subsequently 6-month-old infants, the increase in miR-133b may reflect the increased muscle strength as a result of crawling and walking.

For future cohort studies with multiple time points of measuring saliva miRNAs, chosen biomarkers should be cross-checked with our reported miRNAs known to change over time. If a selected biomarker is known to change over time, age should be included as a covariate. While infant miRNA expression variation was greater than that in adults, age should be controlled in relevant experimental designs.

### 4.10. Endogenous Control miRNAs

Three miRNAs with the lowest coefficients of variation and high prevalences (>95%) across samples were miR-25-3p, miR-16-5p, and miR-23a-3p. Coefficients of variation were 80.9, 84.3, and 84.7, respectively. miR-23a-3p has been previously reported as having a low coefficient of variation in saliva [32]. Out of all three, only one of them accounted for at least 1% of all read counts—miR-16-5p. Additionally, it is only affected by 1 factor: age. So long as this factor is controlled through control participant matching and/or inclusion as covariates, miR-16-5p may be the most suitable internal control miRNA for saliva-based studies.

### 4.11. Limitations

The current study is subject to a few limitations. The number of samples between compared groups were not balanced in all analyses. For example, more samples were collected with RNA stabilizer and swabs compared to without RNA stabilizer and via expectoration. Unequal groups in these comparisons decreased statistical power. Another limitation is that miRNA expression from some samples could have an underlying age bias. For example, samples collected without RNA stabilizer were exclusively collected from children ages 3–10 years old.

## 5. Conclusions

The variables that introduce the largest amount of variation to saliva miRNA biomarker studies are presence/absence of RNA stabilizer and method saliva of collection. Among biologic factors, age is associated with the greatest variation in miRNA levels, particularly in infancy. Studies wishing to control for miRNA expression using a housekeeping gene might consider miR-16-5p, which displays stable abundance in this dataset of 922 healthy individuals. Overall, the current study highlights important factors to consider in future investigations using salivary miRNA expression and emphasizes the need for consistent experimental techniques within individual studies and across study cites.

## Figures and Tables

**Table 1:** Patient Demographics.

	Overall (*n* = 922)
Sex	
Female	435 (47.0%)
Male	473 (51.1%)
Unknown	18 (1.9%)
**Age (years)**	
Mean (SD)	13 (±8.5) ^1^
**Race**	
Asian	21 (2.3%)
Biracial	36 (3.9%)
Black	67 (7.2%)
Hispanic	17 (1.8%)
White	633 (68.4%)
Other	33 (3.6%)
Unknown	119 (12.9%)
**BMI**	
Mean (SD)	23 (±5.3) ^2^

^1^ Age information was unavailable for 8 participants (0.9%). ^2^ BMI information was unavailable for 297 participants (32.1%).

**Table 2:** Sample Characteristics.

	Overall (*n* = 1225)
**RNA Stabilizer**	
Presence	1125 (91.8%)
Absence	100 (8.2%)
**Prandial Status**	
Fasting	58 (4.7%)
Post-Prandial	58 (4.7%)
**Aligner Tool**	
Bowtie1	1225 (100%)
Bowtie2	381 (31.1%)
**Collection Method**	
Swab	991 (80.9%)
Expectorant	234 (19.1%)
**Exercise Status**	
Pre-Exercise	93 (7.6%)
Post-Exercise	93 (7.6%)

**Table 3:** Twenty five miRNAs account for over 80% of all read counts across 1225 saliva samples.

miRNAs	Prevalence	Sum	Median	Variation Coefficient	Percentage of Counts
miR-27b-3p	1169	40,926,803	26,415	94.157	10.479
miR-26a-5p	1166	36,244,335	21,472	102.632	9.280
miR-27a-3p	1154	30,386,731	13,461	119.282	7.781
let-7a-5p	1132	24,298,712	12,728	117.735	6.222
let-7c-5p	1122	20,944,858	8860	133.474	5.363
miR-203a-3p	1162	20,639,200	8841	146.905	5.285
let-7b-5p	1137	19,630,177	6211	143.746	5.026
miR-200a-3p	1108	12,704,002	5658	129.103	3.253
miR-140-5p	855	12,386,415	5632	134.285	3.172
miR-2054	843	11,951,959	4976	138.597	3.060
miR-375-3p	1161	9,732,576	6071	99.767	2.492
miR-203b-5p	1159	9,488,769	5195	125.452	2.430
miR-223-3p	1103	7,058,158	3209	124.497	1.807
miR-205-5p	332	6,941,928	0	251.122	1.777
miR-16-5p	1149	6,791,136	4757	84.338	1.739
let-7f-5p	1135	6,756,344	3727	109.492	1.730
miR-141-3p	873	6,330,255	30	253.150	1.621
miR-22-3p	1162	6,075,567	3065	117.644	1.556
miR-147b-5p	897	4,877,797	2960	114.340	1.249
miR-23b-3p	1143	3,973,076	2566	91.646	1.017
miR-23a-3p	1155	3,833,282	2605	84.696	0.982
miR-21-5p	544	3,783,555	8	309.879	0.969
miR-92a-1-5p	842	3,557,304	2078	113.313	0.911
miR-24-3p	1097	3,393,089	2125	96.327	0.869
let-7g-5p	1108	3,358,980	2056	96.653	0.860

## Data Availability

All data can be found in Table S1.

## Supplementary Materials

The following supporting information can be downloaded at: https://www.mdpi.com/article/10.3390/genes13101874/s1, Table S1: Participant and miRNA Expression Data.
